# Unlocking the bio-economic potential of native non-Apis bee products in Pakistan

**DOI:** 10.3389/fnut.2025.1668237

**Published:** 2026-01-07

**Authors:** Muhammad Adnan Bodlah, Alishbah Mohsin, Ayesha Younas, Aleena Kanwal, Imran Bodlah

**Affiliations:** 1 Center for Insect Farming and Entomological Entrepreneurship (CIFEE), Department of Agricultural Engineering, Khwaja Fareed University of Engineering and Information Technology, Rahim Yar Khan, Pakistan; 2 Department of Entomology, Pir Mehr Ali Shah Arid Agriculture University, Rawalpindi, Pakistan

**Keywords:** antimicrobial resins, bee products, *Ceratina*, *Megachile*, native biodiversity, non-Apis bees, Pakistan, pollination economy

## 1 Introduction

Pakistan's native bee fauna remains scientifically underutilized despite its considerable diversity. Recent surveys have documented 27 non-Apis bee species in the Pothohar Plateau (1) and 11 species in Layyah, Punjab (2, 3), with many belonging to economically significant genera such as *Xylocopa, Megachile*, and *Ceratina*. These solitary and semi-social bees play a vital role in pollination and biodiversity maintenance, especially within native and cultivated ecosystems, yet they remain largely neglected in national research and development frameworks (3).

Globally, non-Apis bees have gained increasing attention for their unique ability to produce bioactive products with considerable therapeutic, nutritional, and commercial value. Their honeys are often richer in antioxidants and antibacterial compounds than Apis honey; their resins exhibit strong antimicrobial and anti-inflammatory properties; and their fermented pollen (bee bread) holds enhanced protein content and probiotic potential (4–6). These characteristics have driven niche industries in Latin America, Southeast Asia, and parts of Africa, where meliponiculture and solitary bee harvesting provide sustainable livelihoods and contribute to the bio-economy (7).

Conversely, Pakistan's natural native non-Apis bee species are biochemically and economically uncharacterized. Despite the presence of suitable habitats and traditional knowledge systems in rural communities, there is minimal awareness, infrastructure, or investment in non-Apis bee product development. Furthermore, ongoing threats such as habitat fragmentation, pesticide exposure, and lack of pollinator-specific policy exacerbate their underutilization. This creates both a scientific knowledge gap and an economic opportunity waiting to be explored (unpublished observations).

The purpose of this paper is to unlock the bio-economy potential of Pakistan's native, non-Apis bees by source recent faunistic surveys, summarize on global research on non-Apis bee products, and recommend ways to work on research, conservation, and commercial aspects related to these bees. This paper supports a multi-disciplinary, sustainability-directed approach to developing bee products that incorporates modern analytical instruments and incorporates the ecological knowledge of local beekeepers. If Pakistan seeks to valorize the whole range of non-Apis bee species that are underused, it can enhance its role in biodiversity conservation and become a major player in the bio-economy of high-value animal products derived from bees. The following sections outline the global relevance of non-Apis bees, describe Pakistan's context and existing research gaps, summarize current knowledge on key bee products, and finally present research, conservation, and policy directions for unlocking their bio-economic potential.

## 2 Native bee products: classes and potential

### 2.1 Honeys and nectars

To date, even though stingless bees (tribe *Meliponini)* have not been reliably found in Pakistan, their family members around the world including *Trigona, Melipona*, and *Scaptotrigona* are known to make honeys with remarkable medicinal characteristics. Honey derived from stingless bees often present 2–3 times the antioxidant capacity and far more potent antibacterial activity than honey produced by *Apis mellifera* due to higher levels of total phenolics, flavonoids, and organic acids (8, 9). For instance, stingless bee honey was shown to inhibit *Staphylococcus aureus, E. coli* and *Pseudomonas aeruginosa* at concentrations below 10 (v/v) which is superior to honey routinely used in clinical settings (10, 11).

Honey produced by stingless bees (*Meliponini*) has multiple properties and uses, including medicinal use, in different regions of the world (12). In Australia, *Trigona carbonaria* honey has high antioxidant activity and has unique physicochemical attributes in comparison to honey produced by *Apis mellifera* (13). *Tetragonula iridipennis* is culturally significant in Nepal, particularly among the Thar people, with 18 known uses relating as food, medicine, heritage, and ideology (14). The stingless bee honey in Ecuador, particularly which from *Scaptotrigona ederi* honey, is prescribed as medicine for wound healing, ocular cataracts, and anti-inflammatory uses. Similarly, honey from 23 species of stingless bees is used as a treatment for cataracts, pterygium, gastritis, ulcers, and wounds in Guatemala, Mexico, and Venezuela (15). All these studies showcase the diverse medicinal applications and cultural significance of stingless bee honey around the world.

Even in Pakistan, locally produced honeys from native flora like *Ziziphus, Brassica*, and *Acacia* species have shown notable antimicrobial zones of inhibition and favorable physicochemical profiles. Although the producers are presumed to be *Apis* species, anecdotal evidence suggests potential involvement of non-Apis bees especially *Ceratina* and *Megachile* which remain under-researched in terms of their nectar processing and honey production potential. Given that such honeys in Southeast Asia and Latin America fetch premium export prices (US $80–120/kg), tapping into this niche could yield considerable economic benefits for Pakistani beekeepers (16–19).

### 2.2 Resins and propolis

Many solitary non-Apis bees, particularly species within *Megachile, Xylocopa*, and *Heriades*, collect resins from local vegetation such as *Prosopis juliflora, Acacia nilotica*, and *Moringa oleifera* to line brood cells or reinforce their nests. These resins are rich in biologically active phenolic acids, diterpenes, flavonoids, and essential oils often displaying antibacterial, antifungal, anti-inflammatory, and antioxidant properties (20, 21).

Propolis from various bee species exhibits diverse bioactive properties. Brazilian propolis contains phenolic compounds like p-coumaric acid and flavonoids, demonstrating strong antifungal activity against Candida species (22). It also shows anti-inflammatory potential through inhibition of cytokines, chemokines, and leukocyte recruitment (23). Stingless bee propolis from Brazil contains phenolic compounds, including the novel finding of resveratrol, and displays antimicrobial activity, particularly against *Candida albicans* (24). A comparative study between Chinese and Brazilian green propolis revealed distinct chemical profiles but similar antioxidant and anti-inflammatory activities (25). These findings highlight propolis as a promising source of bioactive compounds for potential therapeutic applications, particularly in treating fungal infections and inflammatory conditions. However, the chemical composition and bioactivity of propolis vary depending on geographical origin and bee species (26).

While these findings have not yet been replicated in Pakistan, field observations of *Megachile bicollaris* and *Xylocopa aestuans* in Layyah and Pothohar Plateau suggest resin-collection behaviors that merit biochemical exploration (27). Moreover, the use of these resins in traditional wound care by indigenous communities hints at their therapeutic potential, which could be harnessed through scientific validation.

### 2.3 Fermented pollen stores (bee bread)

Unlike *Apis*, which mixes pollen with nectar and enzymes to create bee bread in communal hives, solitary bees like *Ceratina* and Nomia ferment pollen in individual brood cells using naturally occurring lactic acid bacteria (LAB) and yeasts, producing highly nutritious and microbial stable food stores. These stores often show 23%−30% higher protein content, better amino acid balance, and enhanced mineral bioavailability, particularly for iron (Fe), zinc (Zn), and magnesium (Mg) (28, 29).

Recent metagenomic studies have revealed that solitary bee beebread harbors unique strains of probiotic bacteria, including Lactobacillus, Bifidobacterium, and Fructobacillus, which may confer health benefits when consumed by humans (30). In the Pakistani context, *Ceratina smaragdula*, Nomia spp., and *Megachile cephalotes* commonly found in field crops, forests, and orchards could serve as excellent models for characterizing these functional foods. Traditional knowledge in regions such as Khyber Pakhtunkhwa and Gilgit-Baltistan also suggests occasional human use of bee-collected pollen, although these practices are not formally documented or standardized (31).

## 3 Honey bee (*Apis*) and bumble bee (*Bombus*) nest comparison

**Figure 1:**
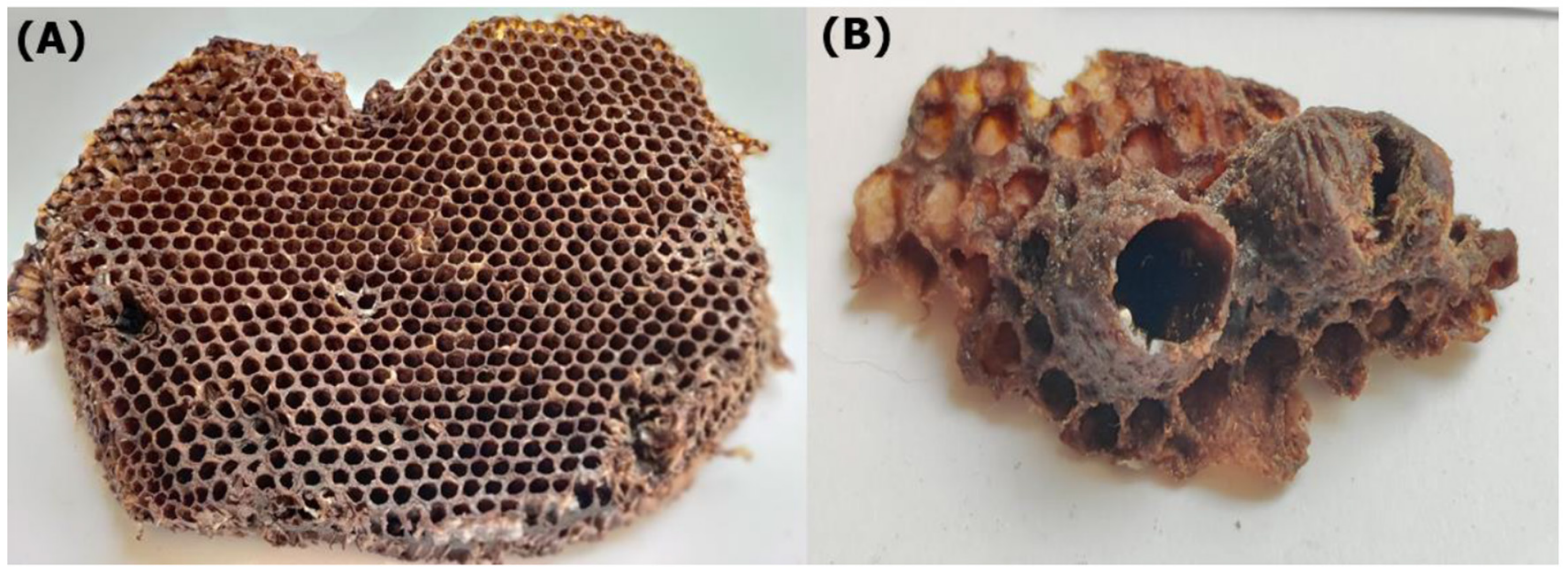
Comparison between the hexagonal-celled combs of honey Bee (*Apis mellifera*) **(A)** and the hemispherical combs (honeypots) of the bumble Bee (*Bombus haemorrhoidalis*) **(B)**.

Honey bees (*Apis mellifera*) and bumble bees (*Bombus* spp.) exhibit distinct comb structures and functional adaptations that reflect their ecological and social organization (Figure 1). Honey bees construct precise hexagonal wax combs arranged vertically for efficient brood rearing and large-scale honey storage (32, 33), whereas bumble bees form irregular, pot-like wax cells often reinforced with plant resins (propolis), enhancing antimicrobial protection (34). These structural differences align with their colony needs honey bees require stable thermal regulation (34–35 °C) for brood development, while bumble bees exhibit less efficient thermoregulation due to smaller, seasonal nests. Worldwide, honey bees dominate commercial beekeeping due to their high-value wax and honey production whereas bumble bees are primarily used for pollination in controlled environments (34–36). In Pakistan, both species contribute to ecosystem services, but honey bees are more extensively managed for apiculture, while wild bumble bees play a vital role in mountain and agricultural pollination despite lacking structured cultivation. The antimicrobial properties of bumble bee combs, enriched with propolis suggest potential biotechnological applications that remain underexplored in the region. While honey bees excel in large-scale resource storage and human utilization, bumble bees demonstrate superior pathogen resistance, highlighting their complementary ecological roles (2, 3, 34).

## 4 Comparison of *Apis* and non-Apis bees biochemicals profiles

Bee products exhibit remarkable biochemical diversity between honeybees (*Apis* spp.) and non-Apis species, with significant implications for their nutritional and therapeutic applications. As earlier discussed, non-Apis bee products consistently demonstrate superior bioactive properties compared to conventional honeybee products (37, 38). Pakistani native species including *Trigona, Megachile, Xylocopa aestuans*, and *Ceratina smaragdula* show particular promise for their enhanced biochemical profiles (unpublished observations).

Non-Apis honeys contain substantially higher antioxidant levels (15–20 mg GAE/g) compared to Apis honeys (5–8 mg GAE/g). In Pakistani stingless bee (*Trigona*) products, which traditional medicine systems have long used for wound healing this boosted antioxidant capability is especially noteworthy. Non-Apis species produce 4,060 phenolic compounds vs. only 2,030 in Apis goods, hence showing even more variance (39).

Resin from *Xylocopa aestuans* in Pakistan's Punjab area exhibits especially rich phenolic variety. Nutritional investigations show non-Apis pollens have 25%−28% protein content compared to 1,822% in Apis pollen (20). Their conventional usage as dietary supplements may be explained by this high protein level found in *Ceratina smaragdula* and Nomia species from the Khyber Pakhtunkhwa province of Pakistan. Against prevalent pathogen, antimicrobial testing reveals non-Apis products create far more extensive zones of inhibition (1,822 mm vs. 10 mm in Apis). Megachile species from northern Pakistan show very powerful activity which is already studied in other regions of the world (40). Perhaps most remarkably, non-Apis bees harbor diverse probiotic lactic acid bacteria (LAB) strains largely absent in Apis products. Additionally, novel bioactive peptides like xylopin (MIC 1.9–15 μM) have been isolated from *Xylocopa aestuans* in Pakistan (41), suggesting untapped potential for drug development.

## 5 Research and development priorities required in Pakistan

### 5.1 Biochemical characterization

Pakistan's non-Apis bees, particularly species like *Xylocopa aestuans, Megachile bicollaris*, and *Ceratina smaragdula*, have not been subjected to rigorous biochemical analysis like many studies on *Apis* species. Profiling their products can provide a foundation for understanding their therapeutic potential. To examine peptide composition of bee-derived products, phenolic, flavonoid, high-performance liquid chromatography-mass spectrometry (HPLCMS) and gas chromatography-mass spectrometry (GCMS) should be used. For instance, resin samples taken from *Xylocopa aestuans* nests can be examined for anti-inflammatory compounds, while fermented pollen from *Ceratina smaragdula* can be tested for probiotic bacteria and nutritional content. Furthermore, against multi-drug resistant pathogens, antibacterial tests including zone of inhibition (ZOI) and minimum inhibitory concentration (MIC) should be conducted (16, 42).

### 5.2 Sustainable utilization

Meliponiculture approaches derived from Brazil and Thailand can be adapted to reflect the nesting behavior of indigenous solitary bees to promote sustainable production. The local community (especially smallholder farmers and women) will require education about safe collection of resin and pollen. The educational methods will stress that seasonal practices and nest preservation techniques can minimize habitat disturbance. Standards for the certification of bee products (including purity, quality, and traceability of source) will improve market access and equitable trading (28, 43, 44).

### 5.3 Conservation and policy

The ecological services of bees other than *Apis* necessitate prompt policy and response action to help protect their habitats, i.e., to include important species such as *Ceratina smaragdula* and *Megachile bicollaris* in national plans for their protection as pollinators. In particular, ground nesting bees require free foraging routes free of pesticides, along with undisturbed soil habitats, and these should be protected! Governments, NGOs, and land managers can use policy and voluntary approaches to ensure bee reserves do exist and that pollinator-friendly farming practices are implemented. Additionally, policy incentives can inspire farmers to preserve flower diversity and limit agricultural chemical overuse (45).

Indigenous non-Apis bees in Pakistan have unrealized potential for pharmacological, ecological, and economic creativity. Their goods might help pollinators, promote rural entrepreneurship, and create a market worth $12 to $15 million annually. Understanding this dream calls for an interdisciplinary approach: combining contemporary chemical analysis with traditional ecological understanding and models of sustainable development. Moreover, it is recommended to prioritize biochemical and quantitative characterization of native non-Apis bee products to scientifically validate their nutritional as well as medicinal potential and support evidence based commercialization. In summary, to strengthen the conservation and sustainable use of non-Apis bees in Pakistan, national programs should be established to support research on species diversity, ecology, and product characterization in collaboration with universities and rural communities. Demonstration units in key ecological regions can facilitate training and technology transfer. Development of regulatory standards for product quality, certification, and market access is essential. Interdisciplinary research linking like chemistry, ecology, agricultural economics, along with integrating traditional knowledge, will enhance the success and sustainability of these initiatives.

## 6 Conclusion

Native non-Apis bee's species of Pakistan represent biologically rich and economically untapped source. However, a systematic documentation of their distribution patterns, product composition and biochemical properties remains largely unknown, highlights a major national research gap. These solitary and semi-social bee species have potential to change the ways in which Pakistan could contribute to traditional medicine, functional foods, and sustainable bio-economy through products such as antimicrobial resins, probiotic bee bread, antioxidant, venom-derived peptides, and bioactive honey. Lack of dedicated research, management approaches, and commercialization pathways is resulting in the underuse of an economically viable resource. So, there is an urgent need of strategic policy to include non-Apis bees within Pakistan's pollinator's conservation framework. By implementing research-led biochemical characterization, sustainable harvesting, and inclusive rural development processes, Pakistan can safeguard its pollinator biodiversity, support new and diverse livelihoods through ecological stewardship processes, and with proper certification, standards, and scientific validation, access cosmopolitan markets with high value products derived from wild native bees. Accessing the potential of native non-Apis bees requires multidisciplinary approaches working across the fields of environmental science, entomology, pharmacology, and rural policy to transform underutilized bee resources into conservation and commercial opportunities. The future efforts should prioritize establishing baseline databases, pilot processing units and clear certification protocols to translate scientific findings into viable policy and market outcomes.

## References

[1] Aslam S Naeem M Hussain S Riasat M Rafi MA Zia A . Biodiversity of non-Apis bees (*Hymenoptera: Apoidea*) in the Potohar region of Pakistan. Diversity. (2024) 17:4. doi: 10.3390/d17010004

[2] Bodlah MA Mohsin A Younas A Hussain S Ashiq A Khan S . Honey bee behavior. in Honey Bees, Beekeeping and Bee Products. London: Taylor and Francis Group (2024). p. 36–52.

[3] Bodlah MA Niaz Y Rasheed MT Fareen AGE Nawaz M Ikram K . Contribution to non-Apis bee fauna of family *Apidae* (*Hymenoptera*) from Layyah, Punjab, Pakistan. Asian J Agric Biol. (2020) 8. doi: 10.35495/ajab.2020.04.227

[4] Amran F Ahmad Zaini MA. On the view of stingless bees' non-honey foods. J Apic Res. (2023) 62:185–202. doi: 10.1080/00218839.2022.2153486

[5] Bakour M Laaroussi H Ousaaid D El Ghouizi A Es-Safi I Mechchate H . Bee bread as a promising source of bioactive molecules and functional properties: an up-to-date review. Antibiotics. (2022) 11:203. doi: 10.3390/antibiotics11020203 35203806 PMC8868279

[6] Khalifa SA Elashal M Kieliszek M Ghazala NE Farag MA Saeed A . Recent insights into chemical and pharmacological studies of bee bread. Trends Food Sci Technol. (2020) 97:300–16. doi: 10.1016/j.tifs.2019.08.021

[7] Rattanawannee A Duangphakdee O. Southeast Asian Meliponiculture. Modern Beekeeping: Bases for Sustainable Production. (2020). p. 173.

[8] Al-Hatamleh MA Boer JC Wilson KL Plebanski M Mohamud R Mustafa MZ. Antioxidant-based medicinal properties of stingless bee products: recent progress and future directions. Biomolecules. (2020) 10:923. doi: 10.3390/biom10060923 32570769 PMC7356725

[9] Rodríguez-Malaver AJ Rasmussen C Gutiérrez MG Gil F Nieves B Vit P. Properties of honey from ten species of Peruvian stingless bees. Nat Prod Commun. (2009) 4:1221–6. doi: 10.1177/1934578X0900400913 19831033

[10] Cruz CBN da Pieri FA Carvalho-Zilse GA Orlandi PP Nunes-Silva CG Leomil L. Antimicrobial activity of honeys from two stingless honeybee species and *Apis mellifera* (*Hymenoptera: Apidae*) against pathogenic microorganisms. Acta Amazonica. (2014) 44:287–90. doi: 10.1590/S0044-59672014000200015

[11] Temaru E Shimura S Amano K Karasawa T. Antibacterial activity of honey from stingless honeybees (*Hymenoptera*; *Apidae*; *Meliponinae*). Polish J Microbiol. (2007) 56:281. 18254500

[12] Silva-Rivera E Vázquez-Domínguez G Mota-Sánchez ÓH. Hernández-De la Cruz I, Franco-José RM, Velázquez-Rosas N, et al. The value of stingless bee bioproducts for human health and conservation: a systematic review. Diversity. (2025) 17:191. doi: 10.3390/d17030191

[13] Oddo LP Heard TA Rodríguez-Malaver A Pérez RA Fernández-Muiño M Sancho MT . Composition and antioxidant activity of *Trigona carbonaria* honey from Australia. J Med Food. (2008) 11:789–94. doi: 10.1089/jmf.2007.0724 19012514

[14] Bhatta C Gonzalez VH Smith D. Traditional uses and relative cultural importance of *Tetragonula iridipennis* (Smith) (*Hymenoptera: Apidae: Meliponini*) in Nepal. J Melittol. (2020) 97:1–13. doi: 10.17161/jom.vi97.13620

[15] Vit P Medina M Eunice Enríquez M. Quality standards for medicinal uses of *Meliponinae* honey in Guatemala, Mexico and Venezuela. Bee World. (2004) 85:2–5. doi: 10.1080/0005772X.2004.11099603

[16] Fahim H Dasti JI Ali I Ahmed S Nadeem M. Physico-chemical analysis and antimicrobial potential of *Apis dorsata, Apis mellifera* and *Ziziphus jujube* honey samples from Pakistan. Asian Pac J Trop Biomed. (2014) 4:633–41. doi: 10.12980/APJTB.4.2014APJTB-2014-0095 25183333 PMC4037657

[17] Gulfraz M Ifftikhar F Asif S Raja GK Asad MJ Imran M . Quality assessment and antimicrobial activity of various honey types of Pakistan. Afr J Biotechnol. (2010) 9:6902–6.

[18] Khan K Ahmad M Ali M Zafar M Haq IU Papini A. et al. Melissopalynological and biochemical profile of honeybee (*Apis mellifera* L.) flora in Southern Khyber Pakhtunkhwa, Pakistan. Plant Biosyst. (2022) 156:1177–86. doi: 10.1080/11263504.2021.2024908

[19] Khan S Khan RU Sultan A Khan M Hayat SU Shahid MS. Evaluating the suitability of maggot meal as a partial substitute of soya bean on the productive traits, digestibility indices and organoleptic properties of broiler meat. Anim Physiol Nutr. (2016) 100:649–56. doi: 10.1111/jpn.12419 26847519

[20] Shanahan M Spivak M. Resin use by stingless bees: a review. Insects. (2021) 12:719. doi: 10.3390/insects12080719 34442285 PMC8397191

[21] Zulhendri F Perera CO Chandrasekaran K Ghosh A Tandean S Abdulah R . Propolis of stingless bees for the development of novel functional food and nutraceutical ingredients: a systematic scoping review of the experimental evidence. J Funct Foods. (2022) 88:104902. doi: 10.1016/j.jff.2021.104902

[22] Freires IA Queiroz VCPP Furletti VF Ikegaki M de Alencar SM Duarte MCT . Chemical composition and antifungal potential of Brazilian propolis against *Candida* spp. J Mycol Med. (2016) 26:122–32. doi: 10.1016/j.mycmed.2016.01.003 26916845

[23] Franchin M Freires IA Lazarini JG Nani BD da Cunha MG Colón DF . The use of Brazilian propolis for discovery and development of novel anti-inflammatory drugs. Eur J Med Chem. (2018) 153:49–55. doi: 10.1016/j.ejmech.2017.06.050 28755848

[24] Rocha VM Portela RW Lacerda LE Sokolonski AR De Souza CO Dos Anjos JP . Propolis from different Brazilian stingless bee species: phenolic composition and antimicrobial activity. Food Prod Process Nutr. (2023) 6. doi: 10.1186/s43014-023-00195-4

[25] Yuan M Yuan XJ Pineda M Liang ZY He J Sun SW . A comparative study between Chinese propolis and Brazilian green propolis: metabolite profile and bioactivity. Food Funct. (2020) 11:2368–79. doi: 10.1039/C9FO02051A 32129351

[26] Izol E Yilmaz MA Gülçin I. Chemical Characterization by Chromatography techniques and comprehensive biological activities of Artvin bee products. ChemistrySelect. (2025) 10:e202501545. doi: 10.1002/slct.202501545

[27] Akram W Sajjad A Ghramh HA Ali M Khan KA. Nesting biology and ecology of a resin bee, *Megachile cephalotes* (*Megachilidae: Hymenoptera*). Insects. (2022) 13:1058. doi: 10.3390/insects13111058 36421961 PMC9698045

[28] Mohammad SM Mahmud-Ab-Rashid NK Zawawi N. Stingless bee-collected pollen (bee bread): chemical and microbiology properties and health benefits. Molecules. (2021) 26:957. doi: 10.3390/molecules26040957 33670262 PMC7917892

[29] Poyraz F Yalmanci D Ispirli H Dertli E. Characterization of bee bread produced with defined starter cultures mimicking the natural fermentation process. Fermentation. (2023) 9:174. doi: 10.3390/fermentation9020174

[30] Graystock P Rehan SM McFrederick QS. Hunting for healthy microbiomes: determining the core microbiomes of *Ceratina, Megalopta*, and *Apis* bees and how they associate with microbes in bee collected pollen. Conserv Genet. (2017) 18:701–11. doi: 10.1007/s10592-017-0937-7

[31] Sher H Aldosari A Ali A de Boer HJ. Indigenous knowledge of folk medicines among tribal minorities in Khyber Pakhtunkhwa, northwestern Pakistan. J Ethnopharmacol. (2015) 166:157–67. doi: 10.1016/j.jep.2015.03.022 25792019

[32] Buchwald R Greenberg AR Breed MD. A biomechanical perspective on beeswax. Am Entomol. (2005) 51:39–41. doi: 10.1093/ae/51.1.39

[33] Yang Y Li W Xie W Wu Q Xu B Wang S . Development of Bradysia odoriphaga (*Diptera: Sciaridae*) as affected by humidity: an age–stage, two-sex, life-table study. Appl Entomol Zool. (2015) 50:3–10. doi: 10.1007/s13355-014-0295-6

[34] Sheikh UAA Ahmad M Aziz MA Imran M Rahim J Roulston T . Rearing of native bumblebee species *Bombus* haemorrhoidalis for greenhouse pollination in Pakistan. Agriculture. (2024) 14:590. doi: 10.3390/agriculture14040590

[35] Evans E. From humble bee to greenhouse pollination workhorse: can we mitigate risks for bumble bees? Bee World. (2017) 94:34–41. doi: 10.1080/0005772X.2017.1290892

[36] Jarimi H Tapia-Brito E Riffat S. A review on thermoregulation techniques in honey bees' (*Apis Mellifera*) beehive microclimate and its similarities to the heating and cooling management in buildings. Fut Cities Environ. (2020) 6. doi: 10.5334/fce.81

[37] Eswaran V Priya V Bhargava HR A. comparative study of the biochemical, antioxidative and anti-microbial activity of *Apis* and *Trigona* honey collected from different geographical areas of India. World Appl Sci J. (2015) 33:160–7.

[38] Zulkhairi Amin FA Sabri S Mohammad SM Ismail M Chan KW Ismail N . Therapeutic properties of stingless bee honey in comparison with European bee honey. Adv Pharmacol Sci. (2018) 2018:6179596. doi: 10.1155/2018/6179596 30687402 PMC6327266

[39] Zaldivar-Ortega AK Cenobio-Galindo A de J Morfin N Aguirre-Álvarez G Campos-Montiel RG Esturau-Escofet N . The physicochemical parameters, phenolic content, and antioxidant activity of honey from stingless bees and *Apis mellifera*: a systematic review and meta-analysis. Antioxidants. (2024) 13:1539. doi: 10.3390/antiox13121539 39765867 PMC11726963

[40] Chuttong B Chanbang Y Sringarm K Burgett M. Physicochemical profiles of stingless bee (*Apidae: Meliponini*) honey from South East Asia (Thailand). Food Chem. (2016) 192:149–55. doi: 10.1016/j.foodchem.2015.06.089 26304332

[41] Goh LPW Molujin AM Muthu K Abdulla R Sabullah MK Mohd Faik AA . Isolation and characterization of lactic acid bacteria from Sabah (North Borneo) stingless bees for probiotic and food applications. Int J Food Prop. (2021) 24:564–78. doi: 10.1080/10942912.2021.1900238

[42] Shafique H Ahmad S Zaidi A. Evaluation of probiotic bacteria isolated from indigenous honeybee species of Pakistan. Lahore Garrison Univ J Life Sci. (2024) 8:79–96. doi: 10.54692/lgujls.2024.0801324

[43] Martins AC Ribeiro TM de A Vasconcelos T. Stingless bees of the amazon forest: taxonomic and geographic gaps and the potential for meliponiculture. bioRxiv. (2025) 2025–07. doi: 10.1101/2025.07.15.664956

[44] Venturieri GC Raiol V de FO Pereira CAB. Avaliação da introdução da criação racional de Melipona fasciculata (*Apidae: Meliponina*), entre os agricultores familiares de Bragança-PA, Brasil. Biota Neotropica. (2003) 3:1–7. doi: 10.1590/S1676-06032003000200003

[45] Byrne A Fitzpatrick Ú. Bee conservation policy at the global, regional and national levels. Apidologie. (2009) 40:194–210. doi: 10.1051/apido/2009017

